# Mutation analysis and prenatal diagnosis of a family with congenital contractural arachnodactyly

**DOI:** 10.1002/mgg3.1638

**Published:** 2021-02-27

**Authors:** Lin Hu, Huanzheng Li, Guang Sun, Ke Wu, Zhaotang Luan, Yanbao Xiang, Shaohua Tang

**Affiliations:** ^1^ Department of Blood Transfusion Second Affiliated Hospital of Soochow University Suzhou China; ^2^ Key Laboratory of Medical Genetics Wenzhou Central Hospital Wenzhou China; ^3^ Department of Clinical Laboratory Yinchuan Women and Children Healthcare Hospital Yinchuan China; ^4^ Prenatal Diganosis Center Yiwu Maternity and Child Health Care Hospital Yiwu China; ^5^ Department of Clinical Laboratory Weifang Hospital of Traditional Chinese Medicine Weifang China

**Keywords:** beals syndrome, congenital contractural arachnodactyly, *FBN2*, prenatal diagnosis, whole‐exome sequencing

## Abstract

**Background:**

Congenital contractural arachnodactyly (CCA) is a rare autosomal dominant condition caused by mutations in the fibrillin 2 gene (*FBN2*). The primary clinical symptoms of CCA include multiple flexion contractures, arachnodactyly, dolichostenomelia, scoliosis, abnormal pinnae, muscular hypoplasia, and crumpled ears.

**Methods:**

We used whole‐exome sequencing technology to examine an arthrogryposis multiplex congenita and used Sanger sequencing technology to genetically confirm its family.

**Results:**

*FBN2* c.3344A>T(p.D1115V) was identified in this family with CCA in a pedigree. Prenatal diagnosis and counseling were carried out simultaneously to avoid the birth of the sick fetus.

**Conclusion:**

The study is on *FBN2* variant in CCA, which potentially having implications for genetic counseling and clinical management, our study may provide new insights into the cause and diagnosis of CCA.

## Graphical abstract

Congenital contractural arachnodactyly (CCA) is a rare autosomal dominant condition caused by mutations in the fibrillin 2 gene (FBN2) Prenatal diagnosis and counselling' were carried out simultaneously to avoid the birth of the sick fetus. We simulated the mutant protein and found that the deletion of the mutation had an effect on the protein.

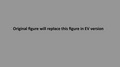

**Abbreviations**
CCA: Congenital contractural arachnodactyly
FBN2: the fibrillin 2 gene
MFS: Marfan syndrome

## 1 INTRODUCTION

Congenital contractural arachnodactyly (CCA, also known as Beals syndrome, or arthrogryposis type 9, OMIM:121050) is a rare, autosomal dominant condition which shares skeletal features with Marfan syndrome (MFS, OMIM:154700). This includes multiple flexion contractures, arachnodactyly, dolichostenomelia, scoliosis, abnormal pinnae, and muscular hypoplasia, but excludes the ocular and cardiovascular complications that characterize MFS cases (Lee et al., 1991; Tuncbilek & Alanay, 2006). While most CCA patients usually have no mental abnormalities, they may have slow motor development (Callewaert et al., 2009). Respectively resulting from mutations in two homologous genes, *FBN2* and *FBN1*, the overlap in clinical features between CCA and MFS has a molecular basis. These highly similar but distinct genes are located at 5q23‐31 and 15q15‐21.3 (Gupta et al., 2002), and encode the large, cysteine‐rich fibrillin‐1 and fibrillin‐2 glycoproteins, respectively (Frédéric et al., 2008). The fibrillin proteins are major components of the extracellular matrix structures called microfibrils, and are found either alone or intimately associated with elastin (Royce & Steinmann, 2003). CCA is a rare disorder with most of the disease‐causing variants identified in the central region, the so‐called neonatal region, of the gene (exons 24–35; Callewaert et al., 2009; Faivre et al., 2009; Gupta et al., 2002; Park et al., 1998; Putnam et al., 1996; You et al., 2017). However, pathogenic variants outside this area have been reported (Callewaert et al., 2009; Meerschaut et al., 2019).

Here, we present a case involving a Chinese family with congenital contractural arachnodactyly which resulted from a novel *FBN2* mutation c. 3344A>T (p.D1115V) identified using whole‐exome sequencing (WES). Sanger sequencing was performed to confirm the presence of the new variant in the other five members from the same family. Once applied, these findings contributed to the pathogenesis of the disease. The disease status of the fetus was simultaneously determined through prenatal diagnosis and counseling. Limited research has been conducted on the application of *FBN2* gene analysis in prenatal diagnosis of CCA. This article gives us a fresh perspective to prevent future cases of CCA altogether.

## 2 MATERIALS AND METHODS

### 2.1 Ethical compliance

All participants provided informed consent, and the study was approved by the ethics committee of Wenzhou Central hospital.

### 2.2 Case presentation

**FIGURE 1:**
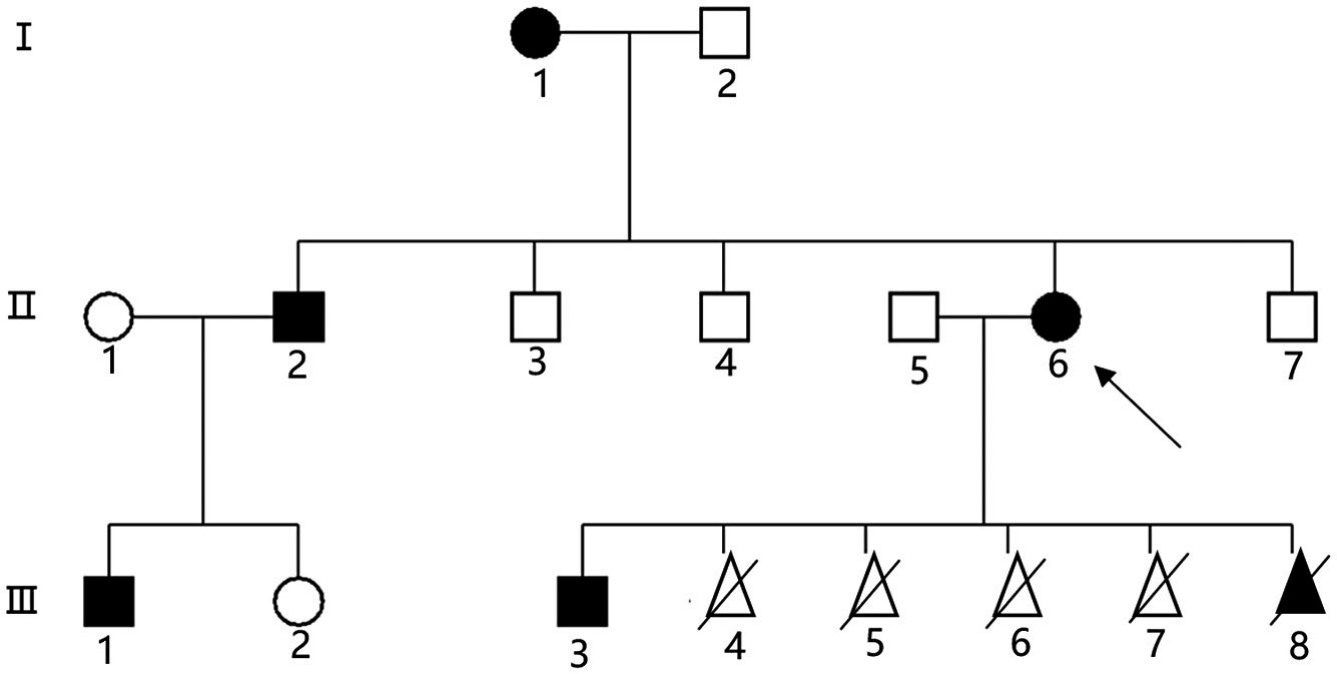
Pedigree of the reported CCA family in this study. Arrow indicates the proband. Black symbols indicate patients with CCA.

**FIGURE 2:**
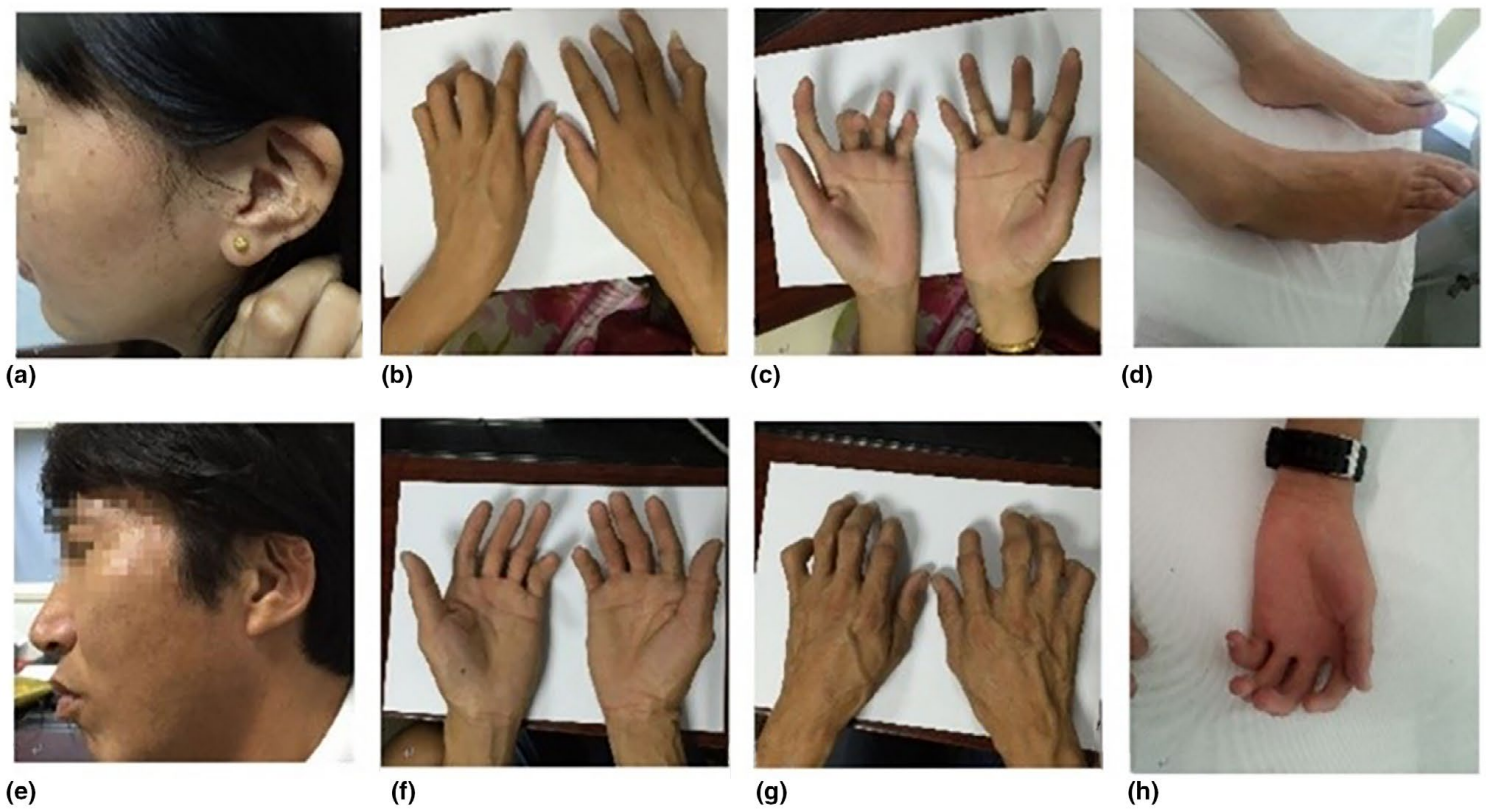
Phenotype of the family. (a) Photographs of ear in the proband; (b,c) Photographs of findings in the proband; (d) Photographs of feet in the proband; (e) Photographs of ear in the II2; (f,g) Photographs of findings in the II2; (h) Photographs of findings in the III3. (h) Photographs of ear in the III3.

We investigate a case of CCA diagnosed in a Chinese family from Zhejiang Province. There are five affected individuals over three generations (Figure 1). Physical examinations of the proband and other family members were conducted to determine the status of CCA. The proband (II6), other affected individuals (I1, II2, III1, and III3), and unaffected individuals (I2, II3, II4, II5, II7, and III2) were simultaneously included in the investigation. All participants had signed informed consent. The proband (II6) was a 39‐year‐old woman with a height of 168 cm and weight of 51 kg. She had long, slim limbs, contractures of the fingers at birth, a pinched, small mouth, crumpled ears, and mild arthrogryposis of the elbows and knees (Figure 2). She visited our prenatal diagnosis clinic at 17 weeks of pregnancy. The other six living members of the family showed the same phenotype. There was no heterogeneity in the phenotype between different genders.

### 2.3 Whole‐exome sequencing and bioinformatic analysis

Blood samples were obtained with informed consent from the family members. At 20 weeks gestation, 40 mL of amniotic fluid was taken under ultrasonic guidance for prenatal diagnosis of the fetus. Genomic DNA was extracted from peripheral blood samples according to the manufacturer's standard procedure using the QIAamp DNA Blood MiNi Kit (Qiagen). WES was performed in the proband.

Variants which have been reported in dbSNP137 or EPS and in Asian populations reflected in the 1000 Genomes Project were filtered and prioritized according to a pathogenicity score >0.95 obtained from Polyphen‐2 (Gan et al., 2012), Mutation Taster (Zhang et al., 2012), and SIFT (Ng, 2003). Variants were additionally cross‐referenced with the Human Gene Mutation Database (HGMD, http://www.hgmd.cf.ac.uk/ac/index.php) and genes known to be implicated in arthrogryposis multiplex congenita were intensively examined.

Primers were designed by using Primer3web (http://primer3.ut.ee/). Using 50 ng of genomic DNA, a 249 bp region of *FBN2* c.3344A>T (NM_001999.4) containing the variant of interest was PCR‐amplified using the *FBN2*‐F (5’‐ AGGTGAAAAGGCACCACTTG‐3ʹ) and *FBN2*‐R (5ʹ‐AGCCACTTTCATAGCCTTCG‐3ʹ) primers. PCR amplification in 50 μL reactions was performed as follows: 95°C for 1 min, 30 cycles at 95°C for 30 s, 58°C for 30 s, 72°C for 30 s, and 72°C for 10 min. The PCR product purification was completed with the E.Z.N.A. ® Gel Extraction Kit. Sanger sequencing was performed on an ABI 3730 DNA Analyzer. Sample preparation for Sanger sequencing was performed according to the BigDye Terminator v3.1 Cycle Sequencing Kit manufacturer's instructions (Applied Biosystems). Evolutionary conservation of the mutant region was investigated with protein sequence alignment generated by the online Clustalw2 tool (http://www.ebi.ac.uk/Tools/msa/clustalw2/).

In this family, amniotic fluid was obtained by trans‐abdominal amniocentesis under ultrasonic guidance at 20 weeks gestation. The analysis of fetal DNA from amniotic fluid was performed to identify disease‐associated variants and perform karyotype analysis.

## 3 RESULTS

### 3.1 Genetic analyses

**FIGURE 3:**
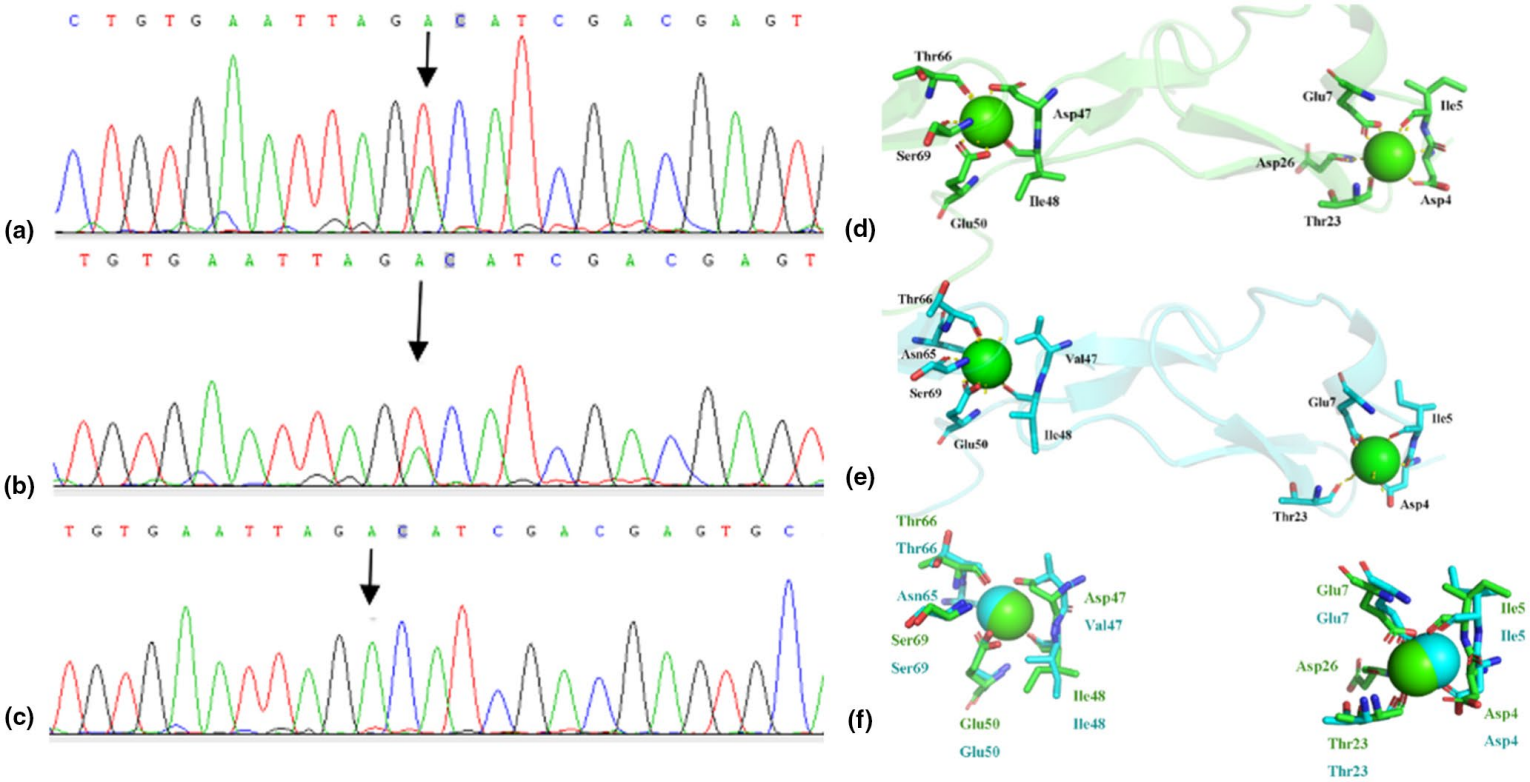
(a) DNA sequencing revealed a heterozygous sing‐base (c.3344A>T, NM_001999.4) affected patients (I1, II2, II6, III1, and III3); (b)fetal amniotic fluid DNA revealed the existence of this mutation;(c) unaffected people (I2, II1, II3, II4, II5, II7, and III2 in this family do not have this mutation. (d) Wild type modeled structure of cbEGF domains 12–13 of the fibrillin‐2, the blue ball means Ca2^+^. (e) Mutant type modeled structure of cbEGF domains 12–13 of the fibrillin‐2, Val 1115 the blue ball means Ca2^+^. The protein modeling is achieved by PyMOL Molecular Graphics System (Version 2.3.0) according to *FBN1* structural coding (Smallridge et al., 2003). (f) Compare wild type modeled structure of cbEGF domains 12–13 of the fibrillin‐2 and mutant type modeled.

In order to identify the genetic cause underlying the CCA cases, DNA from one of the affected family members (II:5, Figure 1) underwent WES. Variants in genes implicated in arthrogryposis multiplex congenita were analyzed. Consistent with the patient's phenotype, a novel heterozygous mutation, c.3344A>T (D1115V), was identified in exon 26 of *FBN2* and was predicted to be pathogenic. This mutation was not found in either the ClinVar database (http://www.ncbi.nlm.nih.gov) or the Human Gene Mutation Database (HGMD, http://www.hgmd.cf.ac.uk/ac/index.php). Confirmed by Sanger sequencing, this mutation was only identified affected family members(I1, II2, II6, III1, and III3) and the fetal amniotic fluid DNA (III8), Unaffected people (I2, II1, II3, II4, II5, II7, and III2) in this family do not have this mutation. (Figure 3). The p.D1115V mutation is a highly conserved amino acid compared to its orthologs and when considered from an evolutionary perspective. SIFT and PolyPhen‐2 predict that the mutation is pathogenic. The variant was classified as likely pathogenic according to the ACMG/AMP 2015 guideline.

### 3.2 Prenatal diagnosis

**FIGURE 4:**
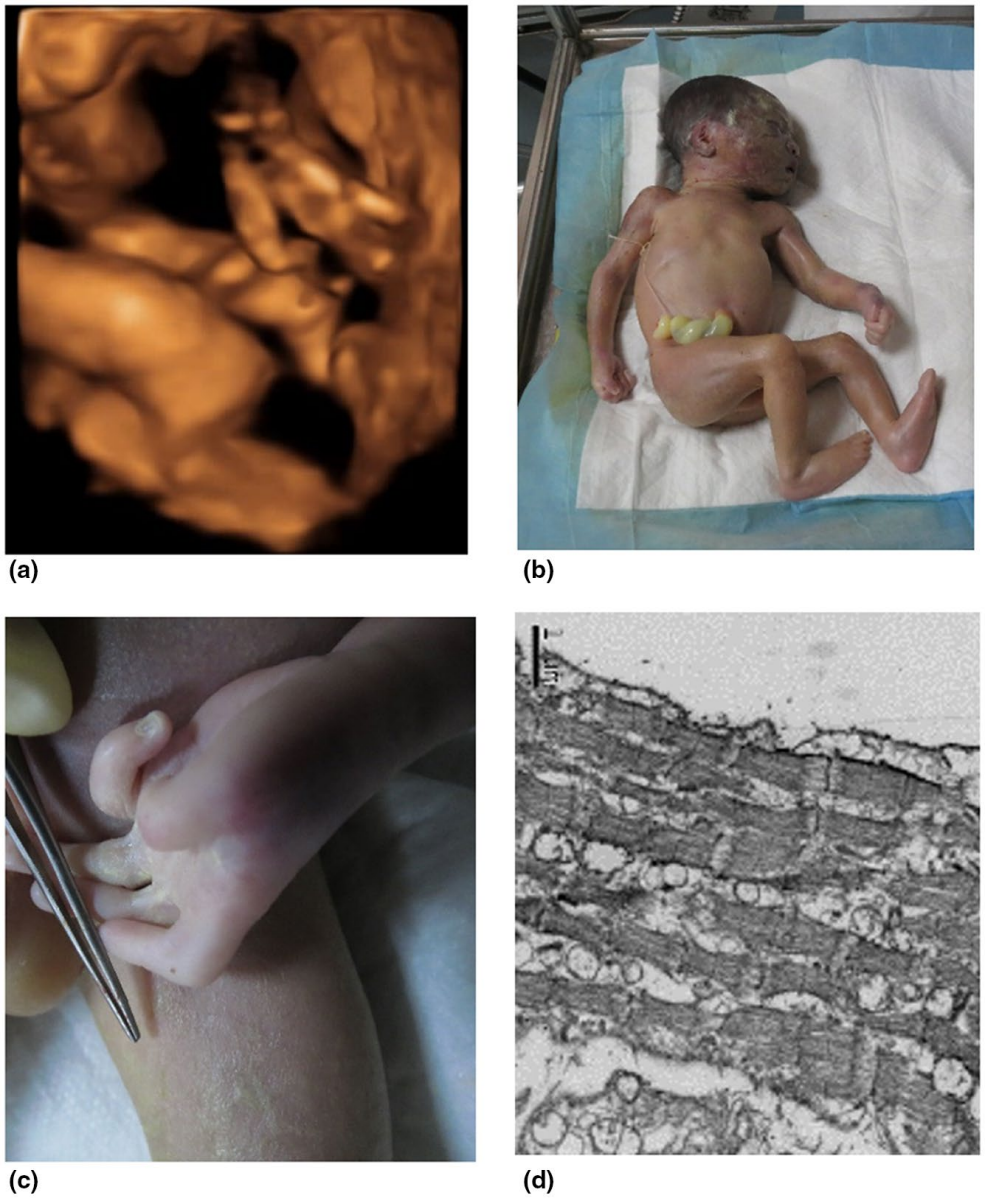
(a) Four‐dimensional B‐ultrasound results showed that when the fetal finger was opened, the finger showed obvious contracture symptoms and could not stretch freely; (b) the picture of the fetus after induction of labor: the fetus hands and feet are abnormal; (c) photograph of the fetal hand: (d) the abnormality of the fetal hand folds, with contracture symptoms; (e) skeletal muscle images of the fetus observed under an electron microscope at 10,000 times.

While the fetal karyotype was normal, sequencing showed that the fetus carried the pathogenic c. 3344A>T (p.D1115V) mutation (Figure 3). Four‐dimensional examination at 29 weeks revealed a male fetus with contracture symptoms. Finger did not show normal extension (Figure 4a), and the double foot position also has an abnormal.

The pregnancy was terminated, and the subsequent pathology showed a slender figure, crumpled ears, mild camptodactly with thick finger joints, and mild arthrogryposis of the elbows and knees (Figure 4b,c). In addition to these, large joints including wrists, elbows, knees, and ankles have more severe contractures. The gastrocnemius electron microscope observed that the bright and dark bands of the muscle segments were not clear, mitochondrial vacuoles appeared between the muscle fibers, and the muscle fibers were loose(Figure 4d).

## 4 DISCUSSION

Congenital contractural arachnodactyly is a rarely autosomal dominant condition caused by mutations in *FBN2*. *FBN2* is the only gene with a known association to CCA (Callewaert et al., 2009), is 279.57 kb in length, encodes a 10,166 bp transcript, and produces a protein comprising 2,912 amino acids. The fibrillin‐2 protein contains five different modules: an EGF‐like domain, calcium‐binding EGF domain, glycine‐rich domain, hybrid domain, and an 8‐cysteine repeat region. Among them, 43 of the 47 EGF‐like domains contain conserved calcium‐binding consensus sequences, which are called cb‐EGF (Ratnapriya et al., 2014). Fibrillin‐2 is limited largely to fetal development (Corson et al., 2004; Zhang et al., 1995). Notably, while contractures may appear mild and tend to reduce in severity postnatally, residual camptodactyly always remains present (Tuncbilek & Alanay, 2006). Similarly, while contracture symptoms in FBN2‐null mice gradually reduced after birth, these mice were associated with a delay in the appearance of a specific perinatal myosin‐8 protein (Sengle et al., 2015). Contrarily, while being highly homologous to *FBN2*, *FBN1* microfibrils act as the regulators for growth factor‐β (TGF‐β) signaling. Dysregulated TGF‐β signaling is associated with MFS (Dieterich et al., 2013; Ochala et al., 2007). The common mutation type of *FBN2* gene is missense mutation, which directly changes the cb‐EGF‐like domain in *FBN2* protein and affects the formation of extracellular matrix microfibers. It is shown in the following three ways: (a) increasing or decreasing Cys residues in cb‐EGF‐like domain, affecting disulfide bond formation and protein folding; (b) change the calcium binding in protein. Sequences and inter‐domain sequences reduce the binding activity of proteins to calcium ions, so that *FBN2* can be hydrolyzed more easily; (c) affect the assembly of various domains, change the spatial conformation and intermolecular interaction of proteins, and so on(Xu et al., 2019).

The c. 3344A>T (p.D1115V) mutation is located in the 26th exon of *FBN2*. There are two mutations located at amino acid 1115, which belong to the highly conserved cbEGF‐like domain, include c.3343G>C (p. Asp1115His) and c.3344G>C (p. Asp1115 Ala) (Callewaert et al., 2009; Meerschaut et al., 2019). This area is belonging to calcium‐binding EGF‐like domain, which is a highly conserved, consists of six conserved cysteine (Cys) residues to form three disulfide bonds to maintain the stability of protein folding in this domain (Ratnapriya et al., 2014; You et al., 2017). Calcium‐binding sequence can directly couple calcium ions or stabilize calcium‐binding sites, which can prevent *FBN2* from being hydrolyzed by hydrolase and play an important role in the interaction between *FBN2* and other components of extracellular matrix.

In *FBN2*, homologous genes of *FBN1* research show that substitution of a histidine at p. Asp1115 position has been shown to abolish its ability to bind calcium (Rao et al., 1995). The c. 3344A>T mutation in *FBN2* was predicted to be deleterious and is conserved in a wide range of organisms ranging from *Anopheles gambiae* to *Pan troglodytes*. This indicates that it might be essential for the normal function of *FBN2*. In addition, we hypothesize that p. Asp1115 may also be pathogenic and could further influence the ability of *FBN2* to bind calcium. After mutating D1115 to V1115, the structural change is more obvious, its RMSD is 0.872, and the position of the calcium ion after the mutation has a slight change, and the hydrogen bond between the calcium ion and the surrounding amino acid has also changed (Figure 4). Missense mutations in *FBN2* gene can also cause amino acid substitutions, such as cysteine (Cys), aspartic acid (Asp), asparagine (Asn), and glycine (Gly). This mutation causes Asp to be replaced, amino acids change from neutral to acidic, changes the side‐chain conformation of amino acid residues, causes adjacent residues to conflict in space, destroys the formation of hydrogen bonds, and affects the stability of protein folding structure. You et al., (2017) found that the mutation in exon 26 c. 3434G > A leads to Gly1145Asp in the 12 cb‐EGF domain in the third generation of Chinese CCA families. A neonatal patient reported c.3973G>A in exon 30 in 2018, resulting in clinical manifestations of p. Asp1325Asn in children with spider‐like fingers, polyarticular contractures, ear shrinkage, minor jaw deformities, and kyphosis (Gurler et al., 2018). The integrity of each domain of the FBN2 protein ensures the stability of the protein, thereby playing its key role in the formation of extracellular microfibers, and participates in the adjustment of the early assembly process of elastic fibers, so that the connective tissue structure remains intact, thereby maintaining human joints The development and function of flexible and normal organs. *FBN2* gene mutation causes abnormal expression of FBN2 protein, destroys the normal structure of connective tissue, and eventually leads to the occurrence of diseases of multiple organs throughout the body. We believe that the mutation disrupted the stability of the protein. Observation of the fetus revealed that the fetus also exhibited symptoms similar to those of the pioneers. After the fetus was induced, it did meet our hypothesis. The clinical manifestations of the fetus were more serious than those of the pioneers and others. Contractile spider fingers, fold ears, elbow joints, and knee joints have obvious contracture symptoms (Figure 4b,c). No obvious abnormalities were found in the general HE staining of fetal skeletal muscles. However, electron microscopic observation of fetal skeletal muscles revealed significant changes in the sarcomere: mitochondrial edema and interfibers. Edema occurred and interfiber looseness (Figure 4d).

Here, we report a novel *FBN2* mutation, c.3344A>T (p. D1115V), in a Chinese family with CCA by use of WES. A prenatal diagnosis and consultation was simultaneously conducted. After inducing delivery of the fetus, contracture of the fingers of the fetus, and the elbow and the knee joints were clearly observed. Observing the muscle tissue through gastrocnemius electron microscopy revealed that the bright and dark bands of the muscle segments were not clear, mitochondrial vacuoles appeared between the muscle fibers, and the muscle fibers were loose.

In summary, whole‐exome sequencing was used to detect the c.3344A>T (D1115V) mutation in the *FBN2* gene of a Chinese family with Congenital Contractual Arachnodactyly. Successful prenatal diagnosis and counseling were conducted simultaneously.

## 5 CONCLUSION

The study is on *FBN2* variant in CCA, which potentially having implications for genetic counseling and clinical management, our study may provide new insights into the cause and diagnosis of CCA.

## CONFLICT OF INTEREST

The authors declare that the research was conducted in the absence of any conflict of interest.

## AUTHOR CONTRIBUTIONS

Lin Hu and Huanzheng Li were involved in the study design, data analysis, and wrote the manuscript. Guang Sun, Zhaotang Luan did the clinical assessment and recruitment of the patients and their family members. Yanbao Xiang and Ke Wu performed the experiments. Shaohua Tang was involved in the study design and critical evaluation of the manuscript.

## Data Availability

Author elects to not share data.
